# Association of fasting blood glucose with global brain macrostructure and AD‐related brain microstructure in non‐diabetic, late‐middle aged males

**DOI:** 10.1002/alz70860_103948

**Published:** 2025-12-23

**Authors:** Jack F.V. Hunt, Jeremy A. Elman, Richard L. Hauger, Christine Fennema‐Notestine, Tyler R. Bell, Erik Buchholz, Carol E. Franz, Matthew S. Panizzon, Chandra A. Reynolds, Rongxiang Tang, Tina T. Vo, William S. Kremen

**Affiliations:** ^1^ University of California San Diego, La Jolla, CA, USA; ^2^ Department of Psychiatry, University of California San Diego, La Jolla, CA, USA; ^3^ Center of Excellence for Stress and Mental Health, VA San Diego Healthcare System, San Diego, CA, USA; ^4^ Veterans Affairs San Diego Healthcare System, La Jolla, CA, USA; ^5^ University of Colorado Boulder, Boulder, CO, USA; ^6^ Texas A&M University, College Station, TX, USA

## Abstract

**Background:**

Diabetes, particularly in midlife, is an established risk factor for cognitive decline and dementia. While clinically defined diabetes represents an advanced manifestation of dysregulated glucose metabolism, even at preclinical levels, elevated blood glucose has been associated with elevated dementia risk. Identifying whether sub‐clinical levels of hyperglycemia in late‐middle age are associated with specific brain structural measures relevant to Alzheimer's disease and related dementia will improve our understanding of links between early stages of dysfunctional glucose metabolism and risk of subsequent cognitive decline.

**Method:**

A sample of 296 non‐diabetic community‐dwelling men (mean age 62, range 56‐65.7) from wave 2 of the Vietnam Era Twin Study of Aging were used for the current analyses. Participants were included if they had completed an MRI scan (T1‐weighted and DTI) and fasting blood draw. Exclusions included major structural brain abnormalities, self‐reported history of diabetes diagnosis, taking diabetes medication or fasting blood glucose (FBG) >125 mg/dL. We assessed associations between FBG and measures of brain macrostructure (global cortical thickness, cortical thickness/volume AD‐signature) and microstructure (global gray matter mean diffusivity, gray matter mean diffusivity AD‐signature, global white matter mean diffusivity and global white matter fractional anisotropy). Waist circumference, fasting HDL and triglycerides and seated blood pressure were measured at the same study visit.

**Result:**

Higher FBG (mean = 96.8 mg/dL, range 69‐122) was significantly associated with lower mean cortical thickness (β=‐0.015, *p* = 0.0051) and higher mean diffusivity in AD‐signature regions (β=0.562, *p* = 0.0386) after controlling for age and other metabolic syndrome factors (waist circumference, fasting HDL, fasting triglycerides and blood pressure). FBG was not significantly associated with the other brain structure measures (*p*'s>0.05).

**Conclusion:**

Higher FBG in non‐diabetic, late‐middle‐aged men without dementia was associated with worse global macrostructural integrity and worse microstructural integrity in AD‐signature regions. This provides evidence that even in preclinical ranges, hyperglycemia may be a risk factor for lower integrity in brain structures relevant to age and cognitive decline independent of other metabolic factors. The results are also consistent with prior evidence that diffusion‐based measures may indicate AD‐related changes earlier than macrostructural measures. Expanding upon these findings into longitudinal studies may help inform clinical and public health efforts.

